# Editorial: Role of Neutrophils in Inflammatory Diseases

**DOI:** 10.3389/fimmu.2020.627939

**Published:** 2020-12-08

**Authors:** Thomas Marichal, Onur Boyman, Laurent L. Reber

**Affiliations:** ^1^ Laboratory of Immunophysiology, Grappe Interdisciplinaire de Génoprotéomique Appliquée (GIGA) Institute, Liège University, Liege, Belgium; ^2^ Faculty of Veterinary Medicine, Liège University, Liege, Belgium; ^3^ WELBIO, Walloon Excellence in Life Sciences and Biotechnology, Wallonia, Belgium; ^4^ Department of Immunology, University Hospital Zurich, Zurich, Switzerland; ^5^ Faculty of Medicine, University of Zurich, Zurich, Switzerland; ^6^ Unit of Antibodies in Therapy and Pathology, UMR1222 INSERM, Institut Pasteur, Paris, France; ^7^ Center for Physiopathology of Toulouse-Purpan (CPTP), UMR 1043, INSERM, CNRS, University of Toulouse, Toulouse, France

**Keywords:** neutrophils, inflammation, neutrophil extracellular trap, immunity, models

Neutrophils are best known for their role in host defense against microbes (1). However, many recent discoveries highlight the fact that neutrophils also act as key regulators of both acute and chronic sterile inflammatory conditions (2). Neutrophils can mediate their functions through different mechanisms, including generation of reactive oxygen species (ROS) and neutrophil extracellular traps (NETs), and the release of a broad range of mediators, including cytokines, alarmins, and proteases (1). A better knowledge of the mechanisms controlling these processes might thus help design new therapeutic strategies for the treatment of inflammatory diseases.

This Research Topic brings together original and review articles that explore the role of neutrophils and neutrophil-derived products in various inflammatory diseases, and assess some molecular mechanisms through which these cells are activated in inflammatory conditions.

It is known that lipopolysaccharide (LPS) from gram negative bacteria can “prime” neutrophil for ROS production in combination with other agonists. Liu et al. used pharmacological approaches to show that the prolyl *cys/trans* isomerase Pin1 plays an important role in LPS-induced priming of human neutrophils. These data suggest that Pin1 could be a therapeutic target in sepsis and other diseases involving ROS generation by neutrophils.

Chiang et al. present an overview of the potential roles of neutrophils in psoriasis, a chronic inflammatory skin disease in which high numbers of neutrophils are observed in skin lesions. Their review focuses mostly on clinical observations, including evidence of neutrophil activation in skin lesions, with generation of ROS and formation of NETs. The authors also discuss the potential contribution of NETs in driving Th17 inflammation in psoriasis.

Lastly, the potential role of NETs in infectious and inflammatory diseases has emerged as a major field of research (3, 4). Not surprisingly, several manuscripts of this special issue focus on the mechanisms of NET formation, and on the role of NETs in various inflammatory conditions. Barbu et al. describe a new method for the detection and quantification of histone H4 citrullination (H4cit3) during NET formation using imaging flow cytometry (IFC). Using this method, the authors quantify H4cit3 in human blood neutrophils stimulated with NET-triggering molecules. They suggest that their method can be particularly suitable for studies looking at NETs as potential biomarkers for diseases. The method is fast and requires only minimal handling of neutrophils, thereby reducing potential unspecific cell activation. However, the authors also emphasize that detection limitations might be associated with some stimuli.

Many infectious and inflammatory diseases are characterized by an increase in vascular permeability. In a review article, Ma et al. present a detailed overview of current knowledge on the potential role of neutrophils in regulating vascular endothelial permeability. The authors particularly focus on the role of NETs and the molecular pathways leading to NET formation, and on the potential role of neutrophil-derived extracellular vesicules, which are heterogenous membrane-enveloped structures released by many cell types, including neutrophils.

Fever is a hallmark of both infectious and inflammatory diseases, and hyperthermia is known to increase neutrophil recruitment to the affected sites. Keitelman et al. studied the impact of hyperthermia on key neutrophil functions including NET release, production of ROS, and release of pro-inflammatory cytokines. The authors propose that fever that occurs during bacterial infections might trigger an emergency response in neutrophils in order to enhance some of their antimicrobial functions, but could at the same time reduce their capacity to release proinflammatory cytokines in order to limit potentially damaging inflammatory processes.

Obama et al. investigate the mechanisms of neutrophil activation in the context of cardiovascular diseases. The authors focused on the effects of oxidatively modified low-density lipoprotein (oxLDL), which are known as a risk factor for the progression of cardiovascular diseases. They show that while oxLDL alone does not induce NET formation, it enhances phorbol myristate acetate (PMA)-induced release of NETs and myeloperoxidase (MPO). The authors suggest that oxLDL may contribute to vascular endothelial inflammation, at least in part by NET-dependent mechanisms.

Granger et al. review the available literature on the role of IgG immune complexes (ICs) in triggering NET release through activation of IgG Fc gamma receptors (Fc*γ*Rs) on neutrophils. They focus on two autoimmune diseases, systemic lupus erythematosus in which NETs can be induced by DNA/anti-DNA IgG ICs, and rheumatoid arthritis for which citrullinated protein/anti-citrullinated protein IgG ICs might mediate NET formation. In addition to autoimmunity, they also review available data on the potential role of ICs-induced NETs in anaphylaxis, a systemic and potentially lethal hypersensitivity reaction. Finally, the authors discuss the potential applications of NETs as biomarkers and therapeutic targets in these diseases.

Most of the aforementioned studies focus on the pro-inflammatory and tissue-damaging potential of neutrophils. However, it was reported that neutrophils also have important regulatory functions, and might participate in the resolution of inflammation (5). In this context, Egholm et al. review existing literature on the regulatory effect of the receptor IL-4R (shared by the cytokines IL-4 and IL-13) on neutrophils during type 2 immune responses. They highlight the fact that engagement of IL-4R on neutrophils can inhibit several neutrophil effector functions, including the formation of NETs. The authors thus propose that, during type 2 immune responses as in other immune responses, neutrophils are the first non-resident cells to arrive at the site of inflammation, but that subsequent IL-4/IL-13 signaling can rapidly shut off neutrophil effector functions to prevent damage to healthy tissues.

Many tools and models are now available to study the role of neutrophils during biological responses *in vivo*. However, each model has its own advantages and limitations. In the review by Stackowicz et al., the authors provide a side-by-side comparison of the main existing genetic and pharmacological approaches to assess the functions of neutrophils and neutrophil-derived products in mice. The authors recommend that, given the known or potential limitation(s) of each model, at least two different approaches should be employed to legitimately conclude on neutrophil functions.

Finally, illustrating the variety of models which can be used to study neutrophils, the work by Degroote et al. focuses on primary neutrophils from equine whole blood. The authors analyzed these neutrophils by mass spectrometry and identified a total of 2,032 proteins forming the whole proteome of these cells. Furthermore, they identified a total of 58 or 207 proteins differentially regulated after stimulation with interleukin-8 (IL-8, also known as CXCL8) or PMA, respectively.

In conclusion, the papers included in this Research Topic highlight the diverse role of neutrophils in the regulation of inflammatory responses, and the potential of future therapies targeting neutrophils or neutrophil-derived products for the management of inflammatory diseases.

## Author Contributions

All authors listed have made a substantial, direct, and intellectual contribution to the work and approved it for publication.

## Funding

TM is supported by the FRFS-WELBIO (Walloon Excellence in Life Sciences and Biotechnology; grant no. CR-2019s-04R), by the Acteria Foundation and by an ERC Starting Grant (grant no. ERC-StG-2018 IM-ID 801823). OB received funding by the Swiss National Science Foundation (310030-172978), the Hochspezialisierte Medizin Schwerpunkt Immunologie (HSM-2-Immunologie), and the Clinical Research Priority Program CYTIMM-Z of the University of Zurich. LR acknowledges support from the French Institut National de la Santé et de la Recherche Médicale (INSERM) and the ATIP-Avenir program.

## Conflict of Interest

The authors declare that the research was conducted in the absence of any commercial or financial relationships that could be construed as a potential conflict of interest.
